# Surgery for Anomalous Aortic Origin of Coronary Arteries: Technical Safeguards and Pitfalls

**DOI:** 10.3389/fcvm.2021.626108

**Published:** 2021-05-12

**Authors:** Massimo A. Padalino, Anusha Jegatheeswaran, David Blitzer, Gabriella Ricciardi, Alvise Guariento

**Affiliations:** ^1^Section of Pediatric and Congenital Cardiac Surgery, Department of Cardio-Thoracic and Vascular Sciences, and Public Health, University of Padova, Medical School, Padua, Italy; ^2^Division of Cardiovascular Surgery, Department of Surgery, Hospital for Sick Children, Toronto, ON, Canada; ^3^Department of Surgery, New York Presbyterian Hospital, Columbia University, New York, NY, United States; ^4^Department of Cardiac Surgery, Leiden Universitair Medisch Centrum, Leiden, Netherlands

**Keywords:** anomalous coronary arteries, surgery, outcomes, techniques, pitfalls

## Abstract

Anomalous aortic origin of a coronary artery (AAOCA) is reported as the second leading cause of sudden cardiac death in otherwise healthy young individuals. Several surgical studies have reported a shallow operative risk, describing repair as safe and effective with short or medium-term follow-up. However, surgical repair can also be associated with a high risk of complications. Numerous repair techniques have been described in the literature, but each technique's indications and limitations are often not well-understood or understated. Since explicit technical knowledge of the most appropriate surgical technique is highly desirable, we sought to thoroughly and clearly outline the safeguards and pitfalls of the most common surgical techniques used to repair AAOCA.

Anomalous aortic origin of a coronary artery (AAOCA) is a congenital heart defect consisting of an abnormal origin and course of a coronary artery that arises from the aorta and differs from the usual pattern (Figure 1A). The most common and clinically relevant anomaly is the anomalous origin from the opposite sinus of Valsalva, including the more common anomalous origin of the right coronary artery from the left aortic sinus (AAORCA, Figure 1B), and the more morbid anomalous origin of the left coronary artery arising from the right aortic sinus (AAOLCA, Figure 1C) (1).

**Figure 1:**
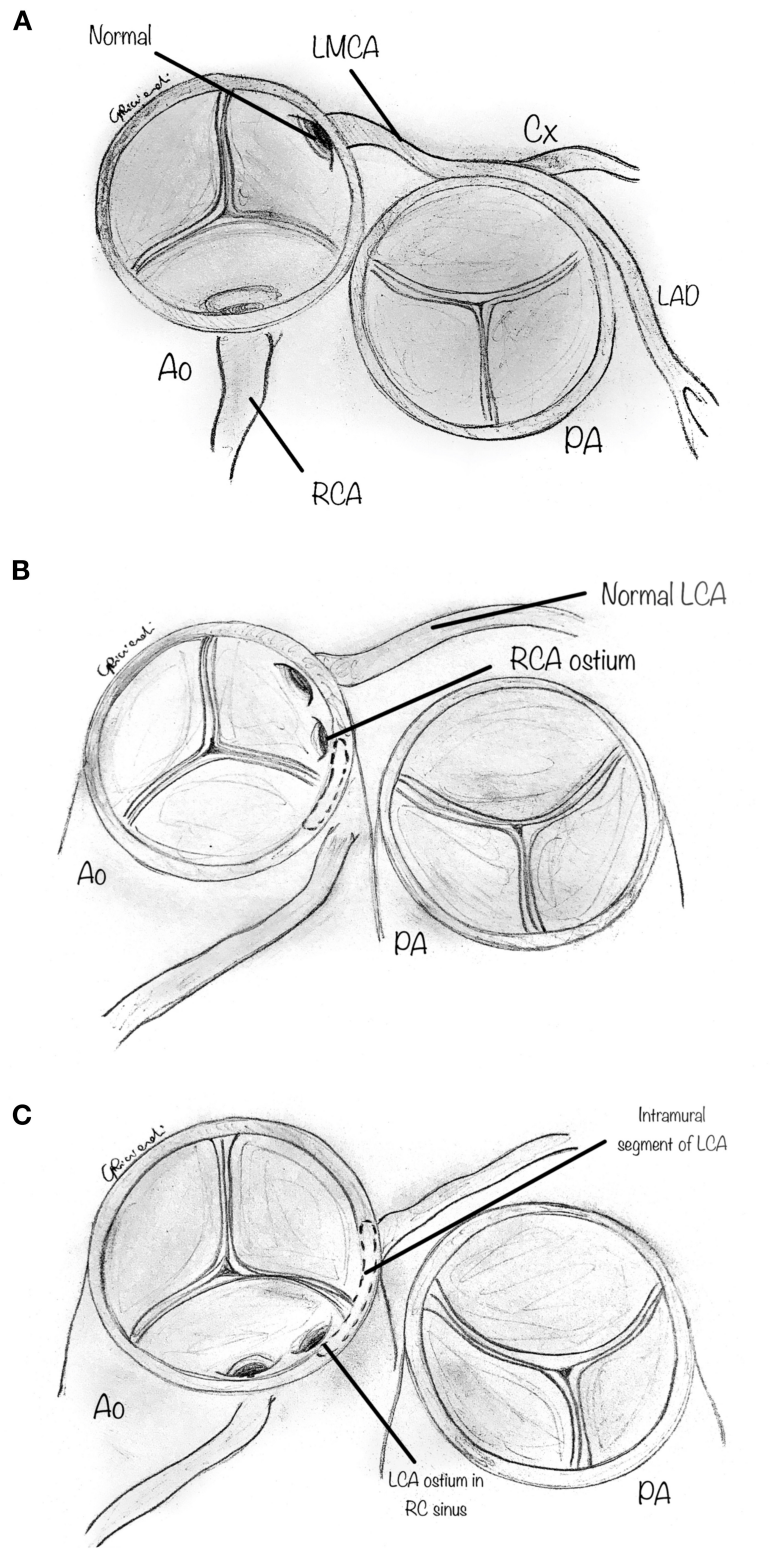
Diagram showing cross section aortic view : **(A)** Normal coronary artery anatomy: the left main coronary artery (LMCA) gives origin to the Left anterior descending (LAD) and circumflex (CX) coronary artery, which arise from a common stem from the left aortic sinus; the right coronary artery (RCA) arises from the right aortic sinus; **(B)** the most common type, i.e., anomalous aortic origin of the RCA from the left sinus (AAORCA), with an intramural segment; **(C)** the most lethal type, i.e., anomalous aortic origin of the LCA from the left sinus (AAOLCA), with an intramural segment.

This congenital anomaly is the second leading cause of sudden cardiac death (SCD) in otherwise healthy young adults (2–4). In the past, the diagnosis was commonly made at autopsy (5). Today, more and more children and young adults are being diagnosed incidentally based on transthoracic echocardiography, computed tomography (CT), or magnetic resonance imaging (MRI) (6). As the exact pathophysiology of AAOCA is still not well-understood, there is a lack of evidence-based guidelines addressing optimal diagnostic work-up, downstream testing, sports counseling, and therapeutic options in patients with such a congenital anomaly (7).

Single-center (8–15) and multi-center studies (16, 17) have reported shallow operative risk, describing repair as safe and effective in the short- and mid-term. Various technical procedures have been applied and reported in the literature, but the necessary minutiae of each technique are often understated, as are the postoperative complications. Emerging data (17) show that coronary-related reoperations and adverse events may occur more often than expected. Given the potentially devastating consequences of unaddressed AAOCA (i.e., SCD), counterbalanced by the risk of iatrogenic coronary complications in the postoperative period, the optimal management strategy is still under debate (18, 19). Currently, surgery is recommended as class I, Level of evidence C, only in patients with AAOCA (either AAOLCA or AAORCA), presenting with typical angina symptoms and with evidence of stress-induced myocardial ischemia in a matching territory, or high-risk coronary anatomy (20).

However, if it is true that high-risk patients should undergo repair of the anomaly when it is discovered, absolute and precise knowledge of the most appropriate surgical technique is highly desirable. Several repair techniques have been described (8, 9, 13, 21–23), but these techniques cannot be universally applied since the optimal surgical maneuver often depends on the patient's coronary anatomy's subtle details.

We sought to describe the most common surgical procedures reported for the repair of AAOCA to outline their safeguards and pitfalls and promote such techniques' optimal utilization.

## Operative Techniques

### Initial Steps

The initial operative steps for all AAOCA repair techniques include standard cannulation for cardiopulmonary bypass. While minimally invasive techniques (reverse T upper mini sternotomy or posterolateral thoracotomy) are probably feasible in straight forward AAOCA, a median sternotomy is often preferred since the risk of the requirement of coronary revascularization can never be ruled out, and more convenient access to internal thoracic artery must be promptly available. After pericardiotomy, careful examination of the coronary pattern can detect all additional external features of the AAOCA. These features should be expected and confirmed in reference to the axial imaging (CT or MRI) obtained preoperatively. The surgeon should note an interarterial course, coronary take-off angle, spatial relationship to the pulmonary artery (PA), and the proximal segment's hypoplasia. A dual-stage venous cannula or bicaval venous cannulation via the superior and the inferior vena cavae can be used, if necessary, for simultaneous intracardiac operations. After aortic and venous cannulation, with full heparinization, cardiopulmonary bypass is initiated, and the cross-clamp is applied. Cold-blood anterograde del Nido Cardioplegia Solution (single shot, which protects the heart up to 180 min) is preferable since it avoids the interruption of repeated dosing during the procedure as is common with other cardioplegic solutions.

### Coronary Unroofing Technique

As shown in Table 1, the most common surgical procedure reported in the literature for AAOCA repair is coronary unroofing (8, 9, 15, 16, 25). This is utilized when a long segment of the proximal coronary course is intramural, with a common wall between the aorta and the coronary artery. Excision of the common wall prevents coronary compression and ischemia during strenuous effort. Otherwise, when blood pressure suddenly increases as occurs with strenuous exercise, the aortic root dilates, and luminal narrowing occurs, reducing coronary perfusion (28).

**Table 1:** Surgical series reported in literature.

**Surgical series**	**Pts n**.	**Age range (years)**	**Technique**	**Early mortality (*n*, %)**	**Early surgical complications (*n*, %)**	**Follow up (years, mean/median)**	**Late cardiac mortality (*n*, %)**	**Late reinterventions (*n*, %)**
Romp et al., 2003 (21)	9	7–65	Unroofing (9)	0	0	2.4	0	1 (11%) Ross operation
Gulati et al., 2007 (22)	18	0.1–16	Unroofing (11) Reimplantation (3) PA translocation (4)	0	0	2.2	0	1 (5.5%) OHT
Frommelt et al., 2011 (9)	27	4–20	Unroofing (27)	0	0	1.8	0	0
Mumtaz et al., 2011 (10)	22	5–54	Unroofing (22)	0	0	1.4	0	0
Wittlieb-Weber et al., 2014 (12)	24	5–18	Unroofing (23) Reimplantation (1)	0	0	5.25	0	0
Sharma et al., 2014 (15)	75	13–70	Unroofing (63) Unroofing + CABG (3) CABG or reimplantation (9)	0	1 (1.3%) ICD	1.56	1 (CVA)	1 (1.3%) AVR
Kooij et al., 2015 (23)	31	9–66	Unroofing (17) Unroofing + reimplantation (8) CABG (1) Ostioplasty (4) Reimplantation +IVS release (1)	0	3 (had VF postop)	6	0	0
Law et al., 2016 (24)	16	17–70	Reimplantation (16)	0	1(CABG + MVR)	5	0	1 (stent)
Mainwaring et al., 2016 (13)	115	0.1–65	Unroofing (86) Reimplantation (9) PA translocation (20)	0	0	6	0	2 (coronary)
Fabozzo et al., 2016 (14)	72	0.1–50.1	Unroofing (64) Reimplantation (8)	0	4 (6%)	1.9	0	1 (AVr)
Nees et al., 2018 (18)	60	0.3–68	Unroofing (56) Reimplantation (4)	0	0	Median 1.6	0	3 (AVR 1, Coronary in 2)ECPR for SCD
Mery et al., 2018 (8)	44	8–18	Unroofing (35) Reimplantation (7) Ostioplasty (1) Side-to-side anastomosis (1)	0	1 (2.2%) CABG	Median 2	0	1 (2.2%) repair of myocardial bridge
Sachdeva et al., 2018 (25)	63	0.5–18	Unroofing (63)	0	0	Median 3.1	1 SCD (2)	0
Ibrahhem et al., 2019 (26)	33	Mean age 34.8 + 4.6	Off Pump CABG with coronary ligation (16)	1 (3%)	0	5.25	1 (3)	1 (redo CABG on pump)
Padalino et al., 2019 (16)	156	15–53	Unroofing (88) Reimplantation (30) Ostioplasty (12) PA translocation (2) CABG (24)	2 (1.3%)	7 (4.5%)	2	3 (1.9)	3 surgical (2.3%)6 (4.5%) interventional
Gaillard et al., 2020 (27)	61	3.7–66.1	Anatomical repair ostioplasty (37) Reimplantation (19) Intraseptal relief (5)	0	(1) ECMO and revision for thrombosis (2) Stenting	3.1	0	1 (revision for patch aneurysm)
Jegatheeswaran et al., 2020 (17)	395	9.9–15.5	Unroofing (334) Reimplantation (24) Ostioplasty (25) PA translocation (22) CABG (3) Other (14)	4/395 (1%)3/395 (CVA)	14	2.8	0	28

*ICD, implantable cardiac defibrillator; AVR, aortic valve replacement; AVr, aortic valve repair; CVA, cerebro-vascular attack; CABG, coronary artery bypass graft; ECMO, extracorporeal membrane oxygenation; ECPR, ECMO cardiopulmonary resuscitation; IVS, interventricular septum; MVR, mitral valve replacement; OHT, orthotopic heart transplantation; PA, pulmonary artery; SCD, sudden cardiac death; VF, ventricular fibrillation*.

**Figure 2:**
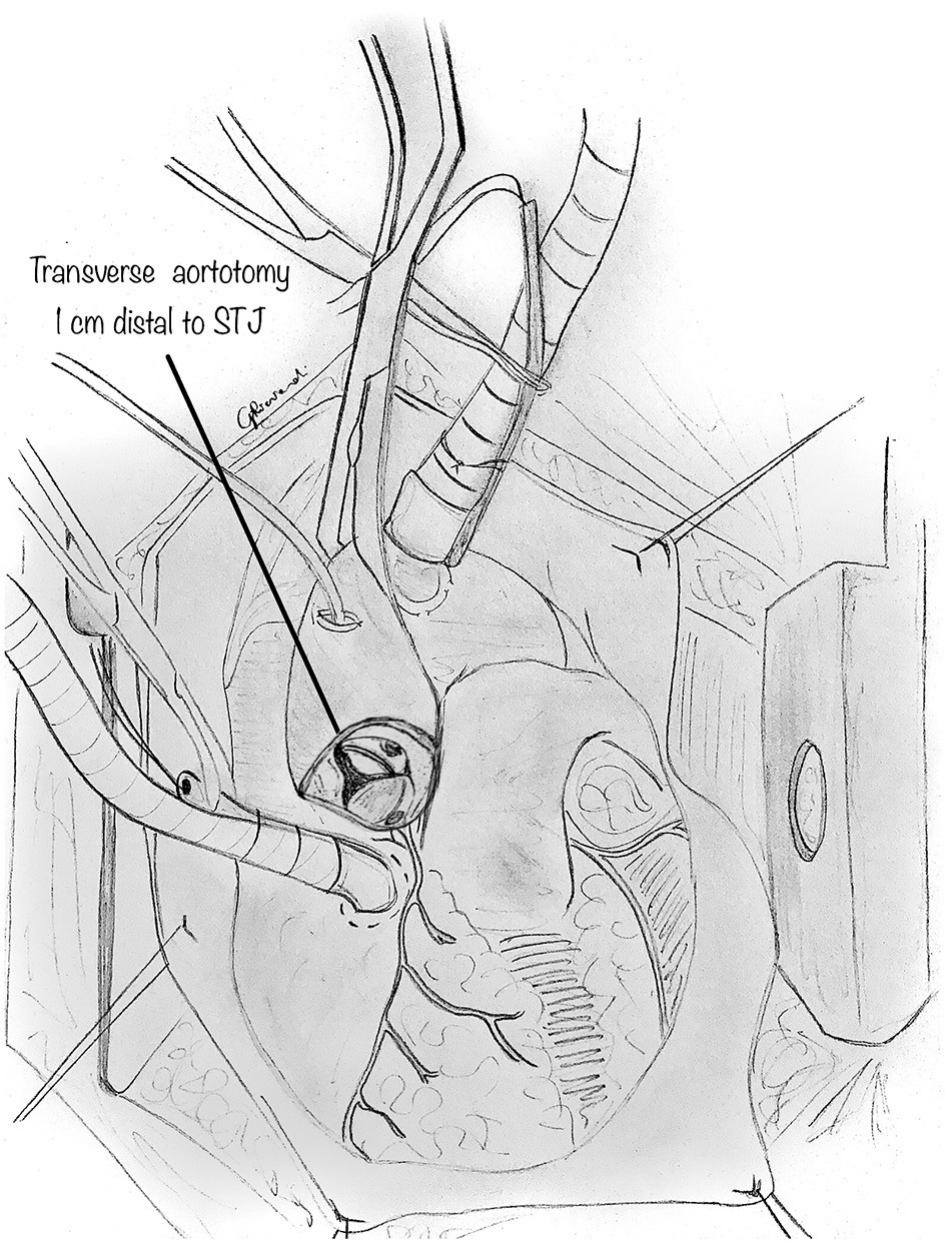
Following a cardioplegic arrest of the heart, a transverse aortotomy is made above the sinotubular junction. The aortotomy is then extended parallel to the annulus of the aortic valve, directed to the left inter-coronary commissure. Additional care must be paid in case of high intramural course of proximal tract of the coronary, to avoid accidental lesions to the anomalous coronary.

After cross-clamping and infusion of the cardioplegic solution, on cardiac arrest, a transverse aortotomy is made above the commissures, about 1 cm distal to the sino-tubular junction (Figure 2). The aortotomy is then extended to the aortic valve's annulus, directed to the left-right intercoronary commissure. Stay sutures are placed in the distal part of the divided ascending aorta to augment the aortic root's visualization. When inspecting the aortic root, the two coronary orifices are usually visualized in the same sinus of Valsalva. In other variations, the coronary origin may be high above the sinotubular junction or may have a common origin with the other coronary artery in the same sinus. In AAOLCA, the right sinus contains a normal-appearing right coronary ostium and usually a tiny slit-like ostium for the left coronary artery. In comparison, for an AAORCA, the left aortic sinus contains a normal-appearing left coronary ostium and a right coronary ostium which may similarly be abnormal.

**Figure 3:**
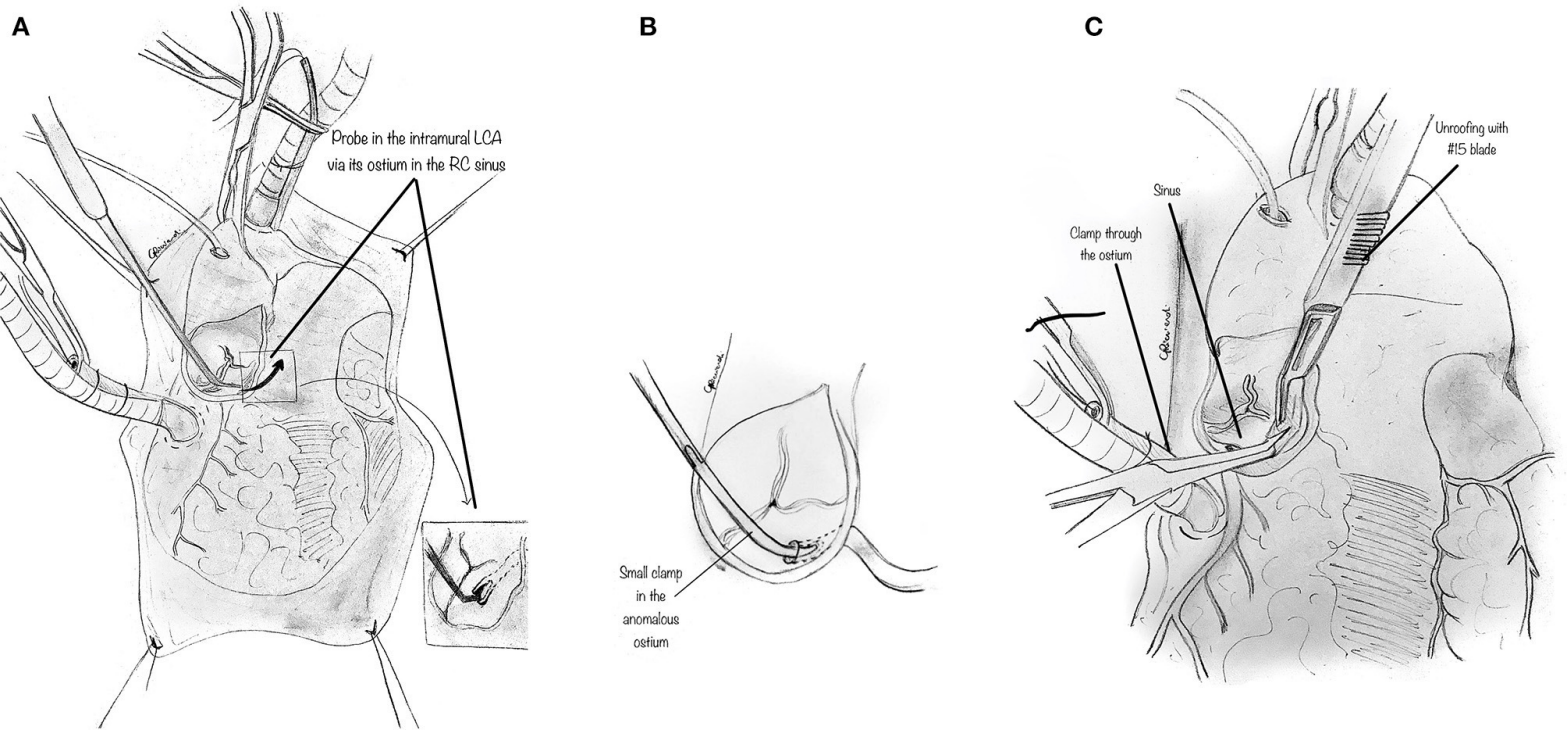
Unroofing technique. **(A)** The orifice of the anomalous coronary artery origin is gently explored with a coronary probe to determine the extent of any intramural segment and its relationship to the correct sinus. **(B)** Diagram showing a small right-angle clamp which is gently placed into the anomalously located coronary ostium to determine its intramural course and evaluate the overlying common wall of the coronary artery and aorta. **(C)** Diagram showing a small right-angle clamp inserted into the anomalously located coronary ostium, and gently pushed inside the intramural course, to reveal the overlying common wall of the coronary artery and aorta which is incised with a blade and unroofed.

The orifice of the anomalous coronary is gently explored with a coronary probe to define an eventual intramural segment's extent and its relationship to the correct sinus (Figure 3A). When the ostia have been identified within the aortic root, a small right-angle clamp can be gently placed into the anomalously located coronary ostium (Figure 3B), and a #15 blade can be safely used to unroof the overlying common wall of the coronary artery and aorta (Figure 3C), along with the intracoronary clamp. The incision may be refined with fine scissors. The target is to incise the common wall until the origin of the anomalous coronary artery from the aorta is exposed, and the coronary exits the aorta from its right sinus.

**Figure 4:**
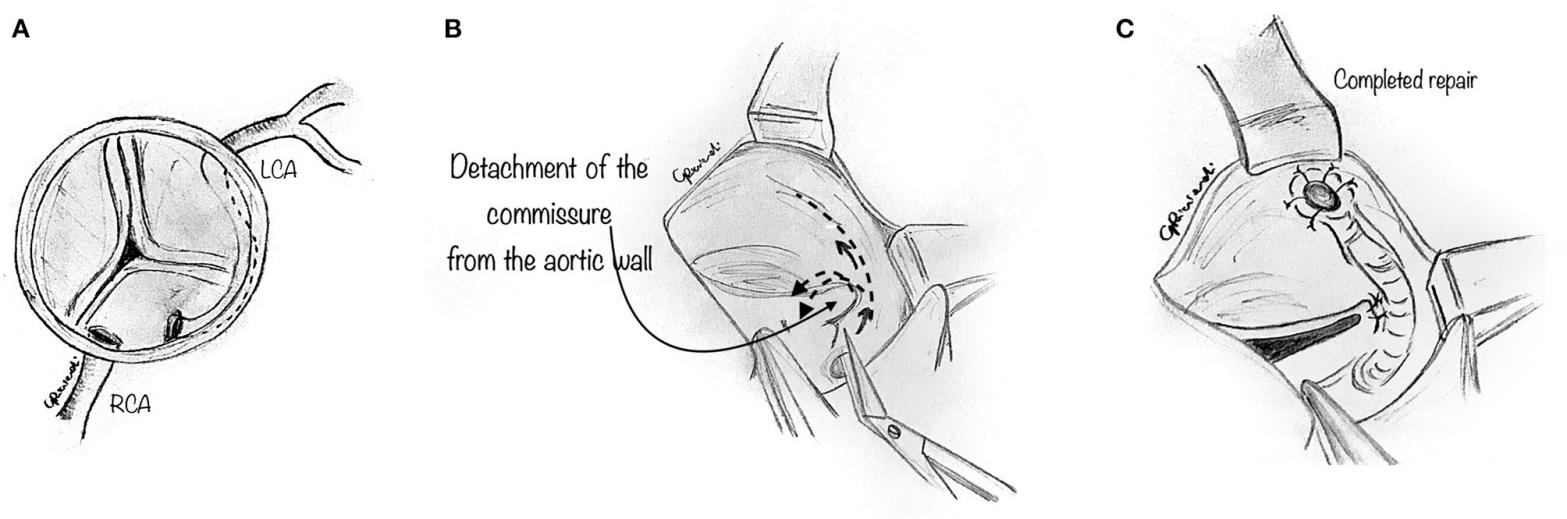
Unroofing technique. The composite figures show a case of AAOLCA in which the intramural portion of the coronary goes from one aortic sinus into the next one by traversing the commissural plane **(A)**. The commissure is gently detached with a blade or fine scissor to expose the intramural portion of the coronary artery **(B)**. Then, the intramural portion of the anomalous coronary is unroofed, by cutting with fine scissors along its course until the take-off of the coronary from the aortic wall. Lastly, the entire intramural course is unroofed and the intima tacked around the new coronary ostium **(C)**.

**Figure 5:**
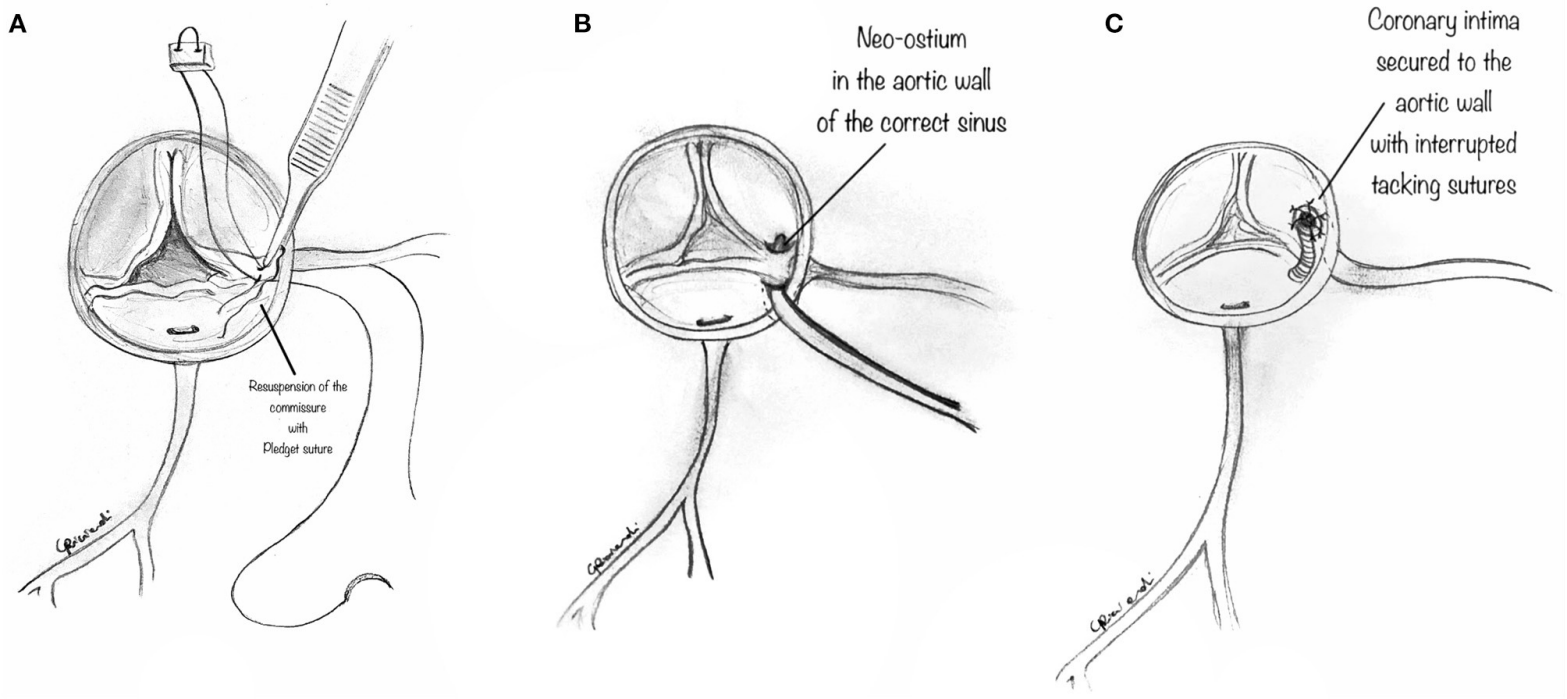
**(A)** The diagram shows that the previously detached commissure is then resuspended with pledgeted sutures in order to create a neo-commissure, and preserve aortic valve integrity. **(B)** The diagram shows a surgical alternative when the intramural coronary segment is located below the commissure. In such a case, a “neo-ostium” can be created in the aortic wall of the correct sinus, opposite to the point where the coronary artery emerges from the aortic wall. **(C)** Interrupted tacking stitches are placed circumferentially around the ostium, securing the coronary intima to the aortic wall with the aim of preventing intimal dissection at the neo-orifice.

In some instances, the intramural portion of the coronary may go from one aortic sinus into the next by traversing the commissural plane, and it is not located entirely above the commissure (Figure 4A). In this situation, the typical unroofing procedure (with the incision of the entire length of the intramural portion) is contraindicated since it would damage the commissure and disrupt the aortic valve leaflets. Thus, the commissure is usually detached, and the intramural portion of the coronary is unroofed by cutting with fine scissors along its course until the coronary take-off from the aortic wall (Figure 4B). Lastly, the entire intramural course is unroofed, and the intima tacked around the new coronary ostium (Figure 4C). Last, the detached commissure is suspended above the unroofed intramural segment with pledgeted suture(s) (Figure 5A). Commissural resuspension is a technique that may lower the rate of aortic valve regurgitation and does not add any proven additional risk to the procedure (29). Alternatively, when the intramural coronary segment courses below the sino-tubular junction, a “neo-ostium” can be created in the correct sinus, at the point where the coronary artery emerges from the aortic wall (Figure 5B). This new ostium is enlarged further, and in this way, the coronary commissure is left intact, leaflet motion is not jeopardized, and a double coronary ostial communication is created. As a result, the angle of take-off of the coronary artery from the aorta is normalized.

After unroofing the intramural segment and creating a neo-ostium in the right sinus of Valsalva, interrupted tacking sutures (7-0 polypropylene) are placed circumferentially around the ostium to secure the coronary intima to the aortic wall to prevent intimal dissection at the neo-orifice (Figure 5C).

A novel unroofing technique has recently been described for adult patients, using electrical cautery to unroof the shared wall tissue along the coronary artery's intramural course (30). With this approach, the surgeon can directly follow the intramural tunnel path and evaporate the aortic wall roof without using a sharp blade to excise the roof of the tunnel. The authors believe that fulguration may be a more straightforward, faster, and better-controlled procedure that can also minimize the risk of potential flap dissection. Also, a wider excision of the shared wall and shorter aortic cross-clamp and cardiopulmonary bypass times are reported. However, a longer and larger follow-up is required to confirm this technique's safety, mainly regarding the potential for coronary endothelial damage that the use of thermal energy can cause.

In conclusion, the unroofing technique presents several advantages. It usually can relocate the functional orifice from the anomalous position to the appropriate sinus. Also, it can enlarge the orifice significantly and eliminate the portion of the vessel that lies between the great arteries. However, the more distal part of the intramural segment, at the point where the coronary artery leaves the aortic wall, is left intact, and the angle of take-off at times may only be minimally normalized. This area may occasionally remain severely stenotic. Thus, an acute take-off angle, the ostium's eccentricity, and hypoplasia of the initial coronary segment may not be adequately addressed by simple unroofing. Also, when detaching and then resuspending the commissure, the aortic valve regurgitation remains at risk. Last, the recent recognition of the intercoronary pillar is of some interest, a thickening of the aortic wall above the commissure, as a possible contributor to both pathophysiology of AAOCA and aortic valve support (29). The intercoronary pillar is the aortic wall segment above the intercoronary commissure, usually thicker than the remaining wall. Usually, the anomalous coronary artery travels from the incorrect sinus and behind and close to this thickened segment. While the unroofing of a long intramural segment can effectively move the ostium into the correct sinus and away from the intercoronary pillar when the intramural segment is short, the simple unroofing may be ineffective since the anomalous coronary artery may be left close to the pillar, and at risk for potential compression. Some authors have reported (29) that the latter can occur after unroofing and suggest alternative surgical techniques in such cases, i.e., coronary translocation. Furthermore, this potential negative outcome may be even more probable when the coronary commissure needs to be resuspended after unroofing of a short segment since this can replace the inter- coronary pillar close to the new coronary ostium.

### Ostioplasty Techniques

An alternative technique for AAOCA with ostial stenosis and the intramural course is extensive coronary ostioplasty, also called “anatomical surgical repair.” As reported by Vouhè et al. (27, 31), the anomalous coronary ostium is completely reconstructed by two incisions, one in the aortic sinus and the other longitudinally on the initial epicardial course of the anomalous coronary artery, which are then joined together.

**Figure 6:**
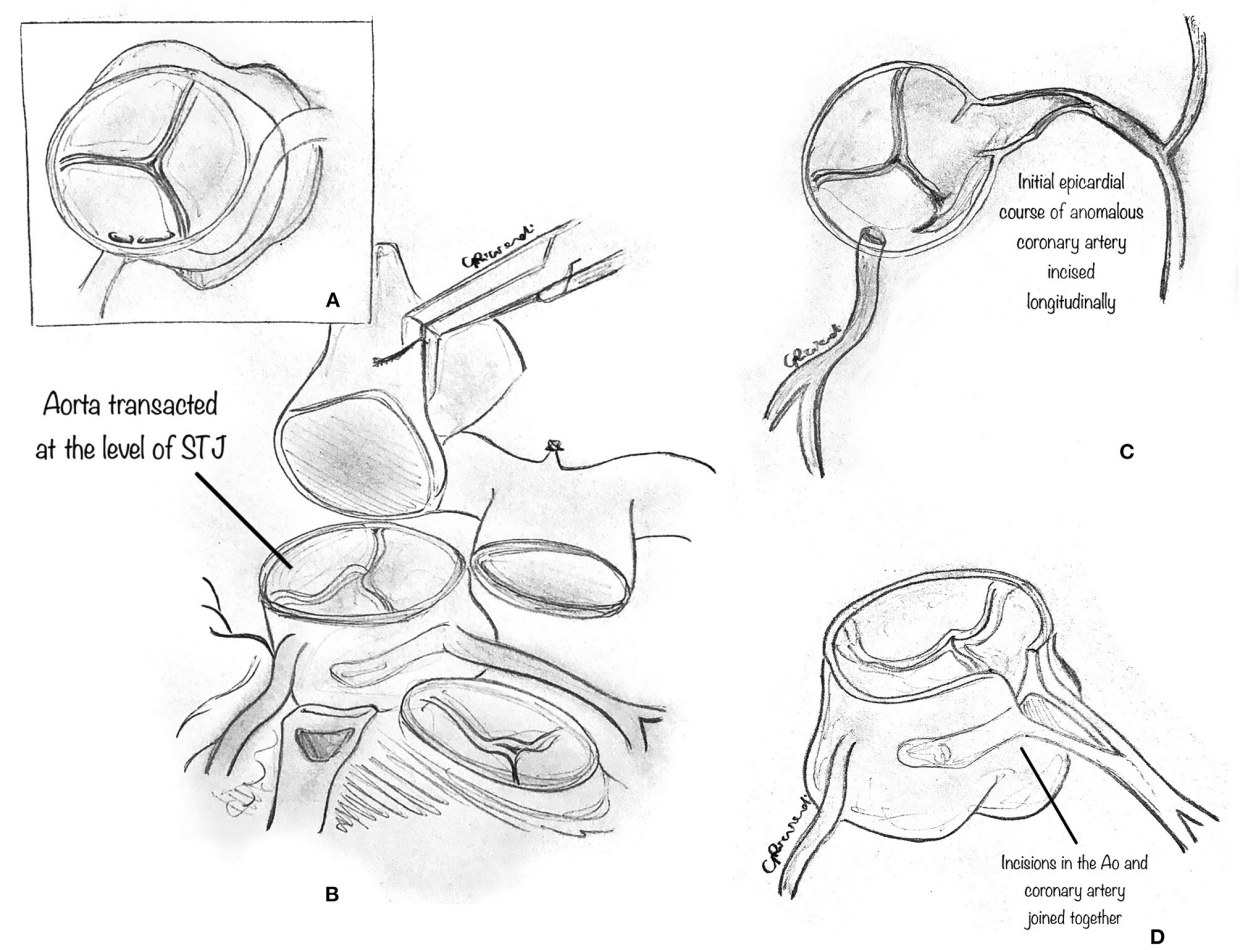
Ostioplasty technique. **(A)** The diagram shows that the aortic and pulmonary roots are separated from each other down to the annular level, to expose the epicardial course of the anomalous coronary artery. **(B)** The normal epicardial course of the left coronary artery is identified and exposed by removing all surrounding connective tissue. The aorta is transected above the sinotubular junction. **(C)** The proximal epicardial course of the anomalous left coronary artery is incised longitudinally. **(D)** The ascending aorta is incised vertically in the left coronary sinus toward the coronary incision, beginning from the cut edge of the aorta. The two incisions, aortic and coronary, are joined at the point where the coronary artery leaves its intramural course to become epicardial.

In detail, the aortic and pulmonary roots are separated (Figure 6A) from each other down to the annular level to expose the epicardial course of the anomalous coronary artery. This step may be facilitated by the division of the PA trunk (31). The normal epicardial course of the left coronary artery is identified and exposed by removing all surrounding connective tissue. Then, the aorta is transected at the sinotubular junction (Figure 6B). The initial epicardial course of the anomalous (left or right) coronary artery is incised longitudinally (Figure 6C). The ascending aorta is incised vertically in the left coronary sinus toward the coronary incision, beginning from the aorta's cut edge. The two incisions, aortic and coronary, are joined at the point where the coronary artery leaves its intramural course to become epicardial (Figure 6D). The intramural segment of the coronary artery is thus left intact but essentially bypassed. Then, a patch of prosthetic material, usually pericardium (although the best patch material to use is yet to be determined), is implanted onto the aortocoronary incision to create a large coronary neo-ostium in the correct aortic sinus. Then, the ascending aorta is reconstructed, incorporating the coronary patch into the anastomotic suture line. Whenever the pulmonary trunk has been transected, it is advisable to reconstruct it after extensive mobilization of the pulmonary arteries. Extreme care must be taken to rule out any residual compression of the reconstructed coronary artery by the PA. Usually, the division of the main PA is not required for AAORCA.

When associated with unroofing, the anatomical repair may be realized with a single incision starting from the cut edge of the ascending aorta, vertically in the coronary sinus, and easily extended in the epicardial course of the coronary artery, which is incised longitudinally.

Anatomical repair with ostioplasty addresses several components of AAOCA: the neo-ostium is enlarged with the patch in the appropriate sinus, with a repair that essentially circumvents the abnormal intramural segment and creates a larger neo-ostium in the appropriate sinus, just at the end of the intramural segment. Doing this, the commissure is left intact, and the risk of iatrogenic aortic insufficiency (due to commissure takedown) may be minimized. According to Vouhè et al. (27, 31), this technique may be effective in most anatomical variants with or without an intramural course, except those with an acute take-off angle or commissural ostial location. The reported early and mid-term results are satisfactory, although the long-term outcomes are still unknown. However, it is a more technically demanding procedure, with a patch in the coronary course, which may thrombose in the long term (27).

One question that remains is which patch material is best. Vouhè et al. (27) suggest a pericardial patch, preferably. However, long-term results are needed to verify what is best. Progressive aneurysmal dilatation of the coronary patch was reported when a saphenous vein patch was utilized, and reoperation was required. An autologous pericardial patch may avoid this complication, as derived from experience with coronary angioplasty in the setting of intramural coronaries in transposition of the great arteries or left main coronary artery atherosclerosis. However, long-term outcomes remain uncertain. Lastly, the potential for late calcification of a pericardial patch, which enlarges the coronary ostium, exists. The use of a prosthetic patch on a coronary artery or ostia is a matter of concern for long-term outcomes. Further and longer follow-up is needed to confirm the safety and long-term effectiveness of this technique.

### Reimplantation

**Figure 7:**
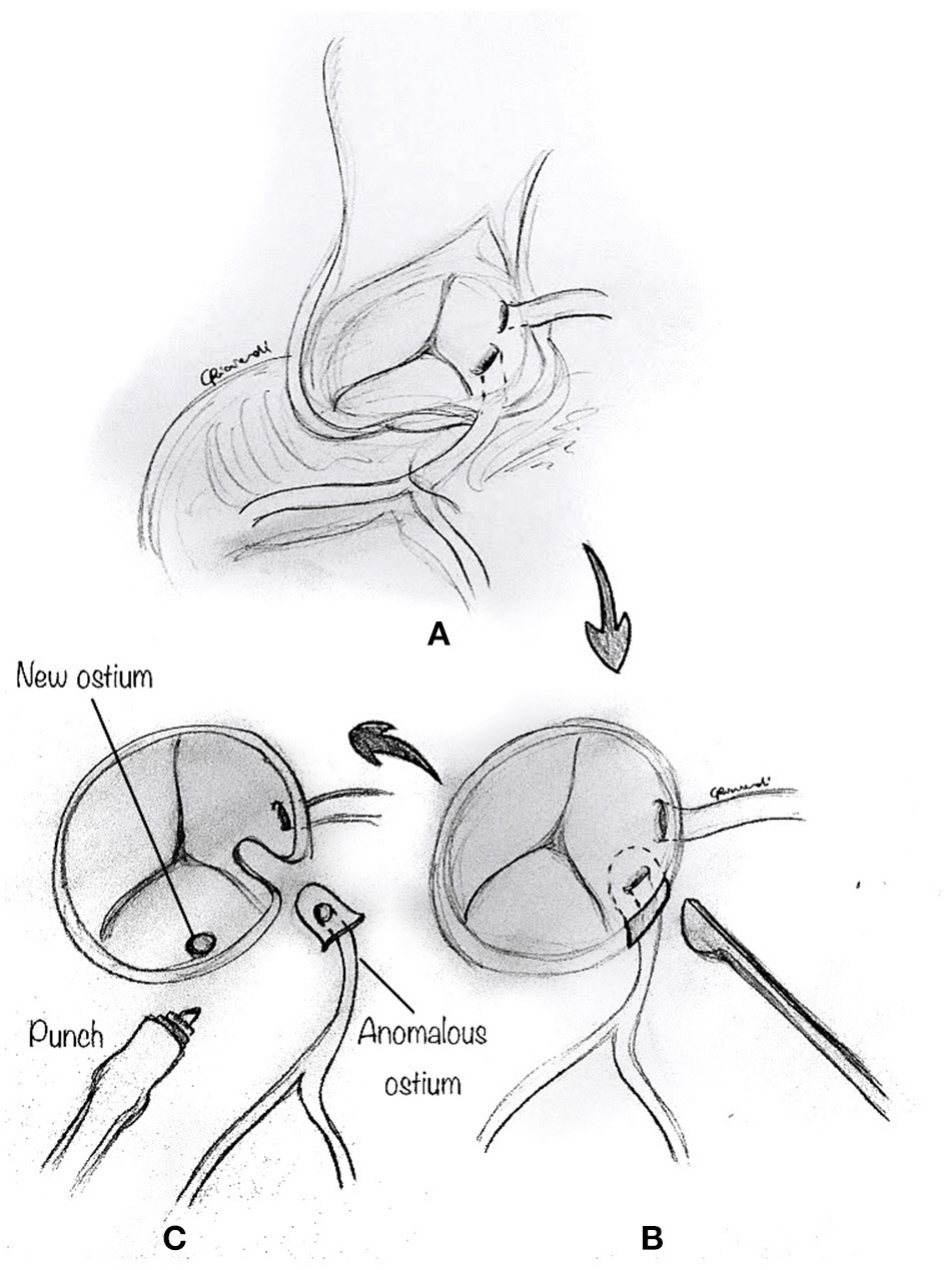
Reimplantation technique. **(A)** The aberrant coronary artery is identified, dissected free along its course, and mobilized by means of low cautery. **(B)** A button of tissue around the ostium of the coronary artery is excised. Attention must be paid to avoid damage to the other coronary ostium, which may be very close. **(C)** A corresponding portion of the correct sinus is then incised with an aortic punch.

**Figure 8:**
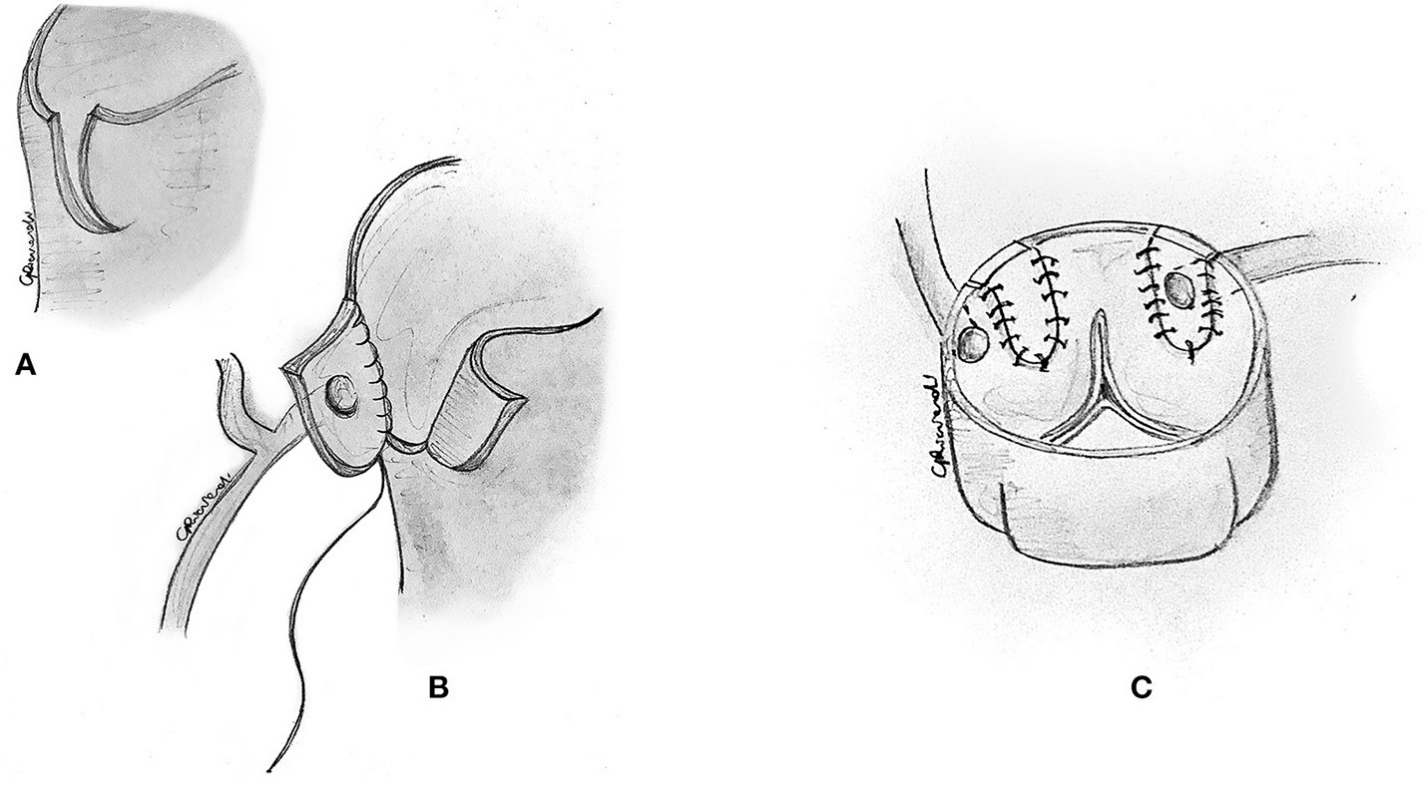
Reimplantation technique. **(A)** Alternatively, a corresponding portion of the correct sinus is incised through a medial trap-door technique. **(B)** The coronary button is sutured to the aorta in its new position. **(C)** The original location of the ostium is usually closed with a small prosthetic patch to reconstruct the aortic wall.

The reimplantation technique is usually reserved for those cases where the anomalous coronary artery course is not intramural, and the coronary arteries have separate origins, or when the commissure is close to the anomalous segment (24, 32). After the usual initial surgical steps, the aberrant coronary artery is identified, dissected free along its course, and mobilized, as commonly performed for an arterial switch operation with a low setting on the cautery. As described above, a transverse aortotomy is performed to better visualize the anomalous coronary artery (Figure 7A). The aortic sinuses are carefully inspected to identify an eccentric location in the sinus, a proximal intramural portion, or a slit-like orifice. Attention must be paid to avoid damage to the other coronary ostium, which may be very close. A button of tissue around the ostium of the coronary artery is excised, as in the arterial switch operation (Figure 7B). A corresponding portion of the correct sinus is then incised either with an aortic punch (Figure 7C) or through a medial trap-door technique (Figure 8A), according to the surgeon's preference. Extreme care is taken to avoid any tension or unnatural kinking of the anomalous coronary artery. The reimplanted coronary position is usually above the correct coronary sinus, distal to the sinotubular junction, to move the coronary artery away from its interarterial course and eliminate the potential for external compression. The coronary button is sutured with 6-0 to 7.0 prolene suture, according to the patient's body weight and surgeon's preference (Figure 8B). The ostium's original location is usually closed with a small prosthetic patch to reconstruct the aortic wall (Figure 8C). Finally, the aortotomy is closed in an end-to-end fashion, with or without reinforcement of the suture line. When the coronary ostium is very close to the commissure, some authors have reported a successful alternative technique in adult patients, consisting of the transection of the anomalous coronary artery at the external aortic exit site, without a button. Then they proceeded to do an end-to-side anastomosis (32).

Selecting the ideal site for reimplantation is crucial in this technique. Coronary reimplantation requires extended coronary mobilization and a perfect repositioning of the coronary in the appropriate sinus to avoid kinking or distortion. When the reimplantation site is too low (whose recognition usually occurs after the bypass is weaned), with suitable ostial enlargement, one can resect a small anterior portion of the aorta above the anastomosis to relocate the right ostium (31) indirectly. This allows adjustment of the effective reimplantation height without the need for resuturing the entire coronary anastomosis.

### Pulmonary Artery Translocation

An alternative approach to AAOCA repair is to translocate the PA away from the aorta, leaving the coronary vessels undisturbed. The principle of the PA translocation (anterior or lateral) is to move the PA away from the aorta and create additional space between the great arteries, reducing the risk of compression of the anomalous coronary artery as it courses between them. This has been demonstrated quite clearly in the postoperative CTA images by Guerra et al. (33), who report an increase in the distance between the PA and the shared virtual origin of the coronaries of about 5 mm. The main advantage of this procedure is that it can be performed without cross-clamping, i.e., on a beating heart.

**Figure 9:**
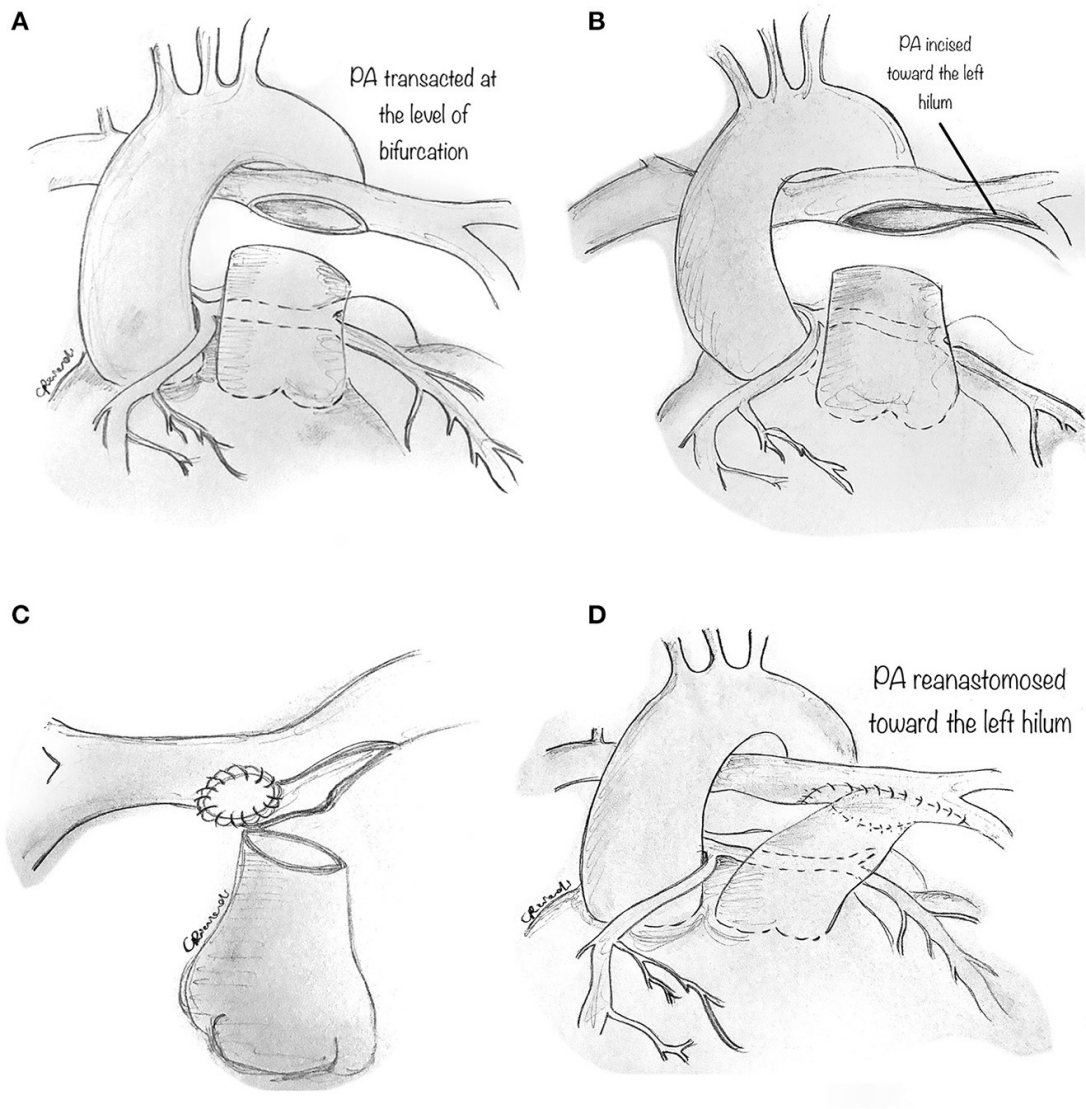
Pulmonary artery translocation technique. **(A)** The perivascular soft tissue between the aorta and the pulmonary artery is debulked. The distal main pulmonary artery is carefully transected at the bifurcation. **(B)** The left pulmonary artery (PA) is incised toward the left hilum. **(C)** A prosthetic material (pulmonary homograft or heterologous pericardium) is then fashioned to widely patch the opening in the PA confluence so as to avoid right PA stenosis. **(D)** The main PA is then re-anastomosed toward the left hilum, resulting in a widely patent PA and pulmonary branches.

Technically, the perivascular soft tissue between the aorta and the PA is debulked. The distal main PA is transected at the bifurcation (Figure 9A), and the left PA is incised toward the left hilum (Figure 9B). A patch of prosthetic material (pulmonary homograft or pericardium) is sutured to enlarge the PA confluence opening and prevent right PA stenosis (Figure 9C). The main PA is then re-anastomosed toward the left hilum, resulting in a widely patent main PA and pulmonary branches (Figure 9D).

Since this technique does not correct the potential causes of coronary hypoperfusion (i.e., slit-like ostium or stenotic intramural segment), we believe it should be utilized in those patients in whom alternatives techniques should be avoided, such as single coronary artery and main coronary artery coursing between the great arteries without an intramural course. Whether this maneuver will eliminate the risk of future coronary compression is not sure since the mechanisms of ischemia in AAOCA go beyond inter-arterial compression. However, PA translocation has some significant advantages: it can be performed on a beating-heart, avoiding myocardial ischemia, and minimal risk of bleeding because of lower PA pressure. As reported and advocated for elsewhere (15), PA translocation is primarily performed as an additional low-risk procedure to other more invasive techniques, such as unroofing or ostioplasty.

### Coronary Artery Bypass Graft (CABG)

**Figure 10:**
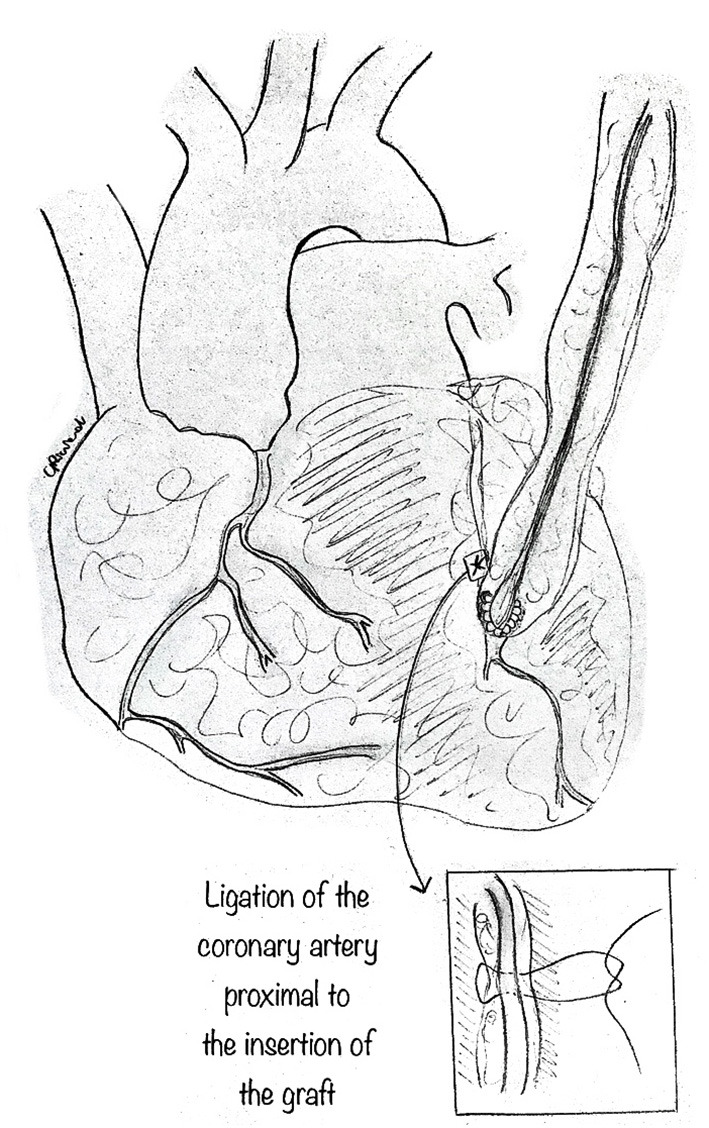
Coronary artery bypass graft. The standard technique can be used, especially in older patients, utilizing the mammary artery as graft to the coronary. In such a case, it is imperative to ligate the coronary artery tract proximal to the insertion of the graft, so as to avoid competitive blood flow which may cause graft.

This traditional technique is performed in the usual fashion (Figure 10). CABG alone may be best suited when other procedures are contraindicated, such as in AAOCA with severe proximal narrowing or in older patients with diffuse atherosclerosis (15, 26), where it shows good early and midterm results. However, CABG, usually with an internal mammary artery bypass, is not recommended in young patients. Since the flow through the anomalous coronary artery is minimally restricted at rest, the mammary bypass may have decreased patency secondary to competitive flow. Fedoruk et al. (34) described 40% late graft occlusion in 5 patients with AAORCA treated with right internal mammary artery graft, while Tavaf-Motamen et al. (35) reported two patients treated with CABG for AAORCA, both of whom had early graft failure with recurrence of symptoms and graft failure. Thus, ligation of the coronary artery proximal to the graft's insertion is an essential step to CABG's success in this setting.

## Discussion

As outlined above, several techniques have been reported to repair AAOCA, but they are heterogeneous and difficult to compare in their effectiveness as different techniques are required based on the different anatomical variations. Besides, indications for surgery are still controversial. While most surgeons still favor repair of AAOLCA even if asymptomatic, repair of asymptomatic AAORCA remains highly controversial (20, 36, 37).

The various surgical techniques are usually devoted to a particular anatomical subtype of coronary anomaly and only deal with 1 or 2 pathophysiological mechanisms of hypoperfusion (5). Ideally, the coronary surgical reconstruction for AAOCA should normalize the anatomy, relocating a large ostium in the center of the appropriate sinus, reproducing a normal take-off angle, and eliminating any intramural or interarterial course. As a matter of fact, none of the techniques described above can address all of these components, and each is susceptible to individual technical pitfalls. Also, the use of a prosthetic patch on a coronary artery or ostia is a matter of concern for long-term outcomes.

To summarize, the following techniques should be considered as the repair for these specific issues.

Unroofing is indicated to manage an intramural course. However, it can be limited by the commissure location, leading to the requirement for a commissural resuspension. Ostioplasty is primarily utilized to manage a small or stenotic coronary origin. The anatomical repair technique (31) and the recently described “unflooring” technique (that includes a patch augmentation of the intramural course in addition to standard unroofing) (38) imply an increasing technical effort aimed at optimizing the clinical outcomes. While it eliminates the inter-arterial course and the entire intramural segment and augments the coronary ostium by a pericardial patch plasty, a limitation of this procedure is that the exit from the aorta is very eccentric, typically with a very acute take-off angle that may be difficult to correct.

Furthermore, a prosthetic patch is utilized, with the potential of possible late ostial stenosis. Coronary reimplantation is to be favored when the intramural segment is too short or absent, and adjunct PA is used to increase the space around the coronary artery potentially. Last, PA translocation may be preferentially utilized as an additional low-risk procedure in an attempt to improve the outcomes. As such, a mixture of different surgical alternatives can be utilized to better repair AAOCA.

## Conclusions

A thorough understanding of coronary pathology and applying the appropriate operative techniques are critical to achieving excellent results for both adult and pediatric patients. A potential trend toward minimally invasive approaches to the surgical repair of AAOCA may be promising and could further improve quality of life. Investigations of long-term outcomes and a more comprehensive understanding of this lesion's natural history and its variations remain imperative.

## Author Contributions

MP conceptualized, wrote, and revised the paper, coordinated the team, and supervised the authors. AJ and AG conceptualized and revised the paper. DB revised literature and revised the paper. GR revised the paper and produced the artwork. All authors contributed to the article and approved the submitted version.

## Conflict of Interest

The authors declare that the research was conducted in the absence of any commercial or financial relationships that could be construed as a potential conflict of interest.
